# A Case of Isolated Bilateral Femoral Bifurcation in Saudi Arabia

**DOI:** 10.7759/cureus.33816

**Published:** 2023-01-16

**Authors:** Ghadeer T Alelawi, Areej M AlNassir, Malak T Bukhamsin, Malak O AlDossary, Ismail H Almogbil

**Affiliations:** 1 General Practice, King Fahad Hospital, Riyadh, SAU; 2 Medicine, King Abdullah Bin Abdulaziz University Hospital, Riyadh, SAU; 3 Anesthesia, Health Cluster Eastern Province, Dammam, SAU; 4 Anesthesia, Maternity and Children Hospital, Riyadh, SAU; 5 Surgery, Unaizah College of Medicine and Medical Sciences, Qassim University, Buraidah, SAU

**Keywords:** bifurcation, developmental anomaly, bifid femur, bilateral, distal femur, congenital, dysgenesis

## Abstract

Bifurcation of the femur is a rare congenital orthopedic anomaly that is usually accompanied by tibial agenesis. Femoral bifurcation has been a part of the Gollop-Wolfgang complex criteria, which also include the tibia and fibula deformities with or without other variant abnormalities. Approximately 200 cases have been reported worldwide. This article presents a case of isolated bilateral distal femur bifurcation in a three-and-a-half-year-old girl who presented with abnormal gait and bilateral knee contractures. Extensive studies were performed to aid an appropriate diagnosis and treatment plan, including clinical history, physical examination, laboratory, and other investigations. After multiple consultations from different specialties, a gradual deformity correction was suggested as a treatment of choice. To our knowledge, there is a paucity of reported cases in the medical literature. Further research and case reporting are essential to better define the pathogenesis, prognosis, and treatment of such conditions.

## Introduction

Within the last few decades, hundreds of cases with congenital anomalies of the femur have been reported. Distal femur bifurcation is a rare congenital anomaly and is always accompanied by deformity or aplasia of one of the lower leg bones [1]. In 1885, Ehrlich was the first to describe femur bifurcation in a tibial hypoplasia-related patient, and since then, the same defect has been reported [2]. Although the two disorders can occur separately, they usually co-exist and are accompanied by additional congenital malformations of the limbs or other body parts [2]. Habou et al. described a disease called the Gollop-Wolfgang complex, which is characterized by distal femoral duplication and tibial hypoplasia with or without hand ectrodactyly [3]. However, an isolated finding of distal femur bifurcation was only reported once in the late 80s [1]. Based on our search through the literature, no reported cases in Saudi Arabia have been found.

## Case presentation

In late August 2021, a three-and-a-half-year-old female presented to the outpatient clinic with an abnormal gait since she took her first steps. The mother noticed it early and described it as a “flexed knee walk.” There was a growing concern from the parents when the patient complained of pain at the knee site in addition to a lump sensation laterally. The patient was born of non-consanguineous parents and premature labor (32 weeks) due to preeclampsia with a history of admission in the neonatal intensive care unit because of sepsis and grade one intraventricular hemorrhage. During her lifetime, there were no observed abnormalities or mental, developmental, or other musculoskeletal concerns. The family sought medical advice multiple times before this visit. At the age of 19 months, a hamstring tenotomy was performed bilaterally, followed by a specialized physiotherapy program. Upon clinical examination, the girl was standing and walking with bilateral flexion contracture of the knees with no functional limitation. A general lower limb examination showed normal neuro-vascularity with a normal range of motion except for the knees. They appeared relatively larger compared to her overall body, with contracture at approximately 45 degrees, and grossly intact skin. On palpation, there was no joint line tenderness but an irregular bony swelling was noted laterally on both sides. Moreover, there were no positive specific tests and no signs of inflammation or systemic involvement. Further, extensive investigations performed for the past year included complete blood count, white cell differential, and inflammatory markers, which were appropriate to the serum levels of her age group. In addition, minerals such as calcium, phosphate, potassium iron, and vitamin D were within acceptable ranges.

**Figure 1:**
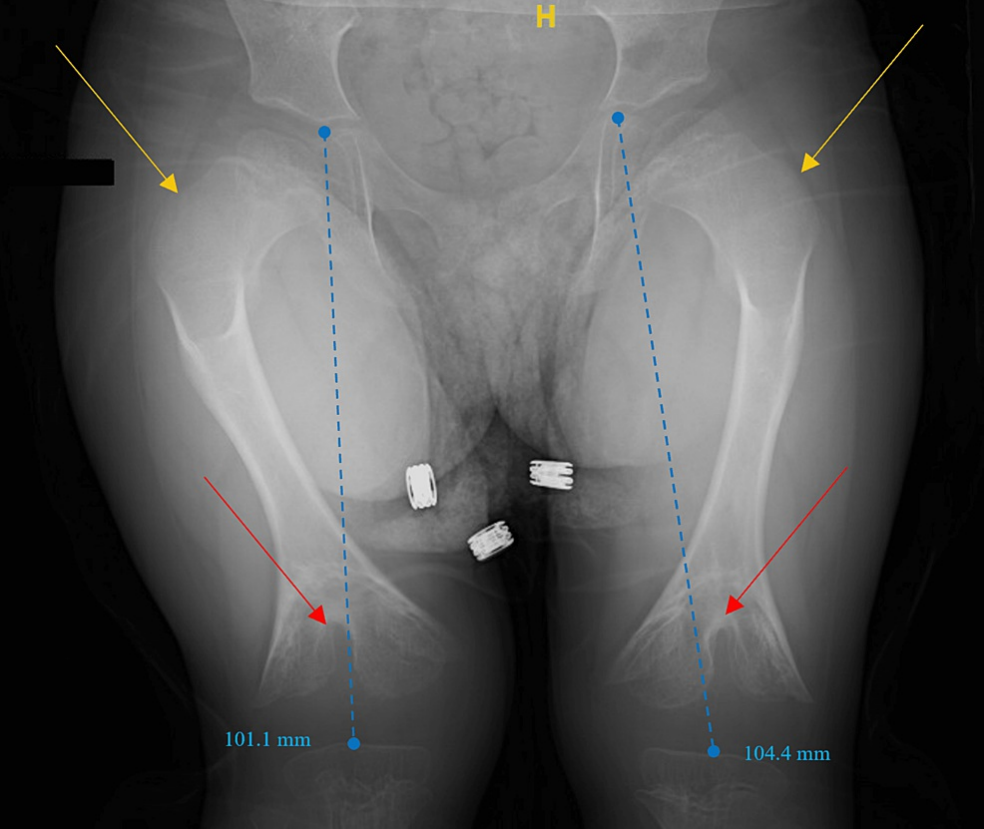
An X-ray of the bilateral lower limbs (anteroposterior view) was performed in December 2021. In this figure, the colors illustrate the following: blue: femoral shortening; red: cone-shaped deformity of distal femur metaphysis with a central fusion of the epiphysis; yellow: femoral neck widening.

**Figure 2:**
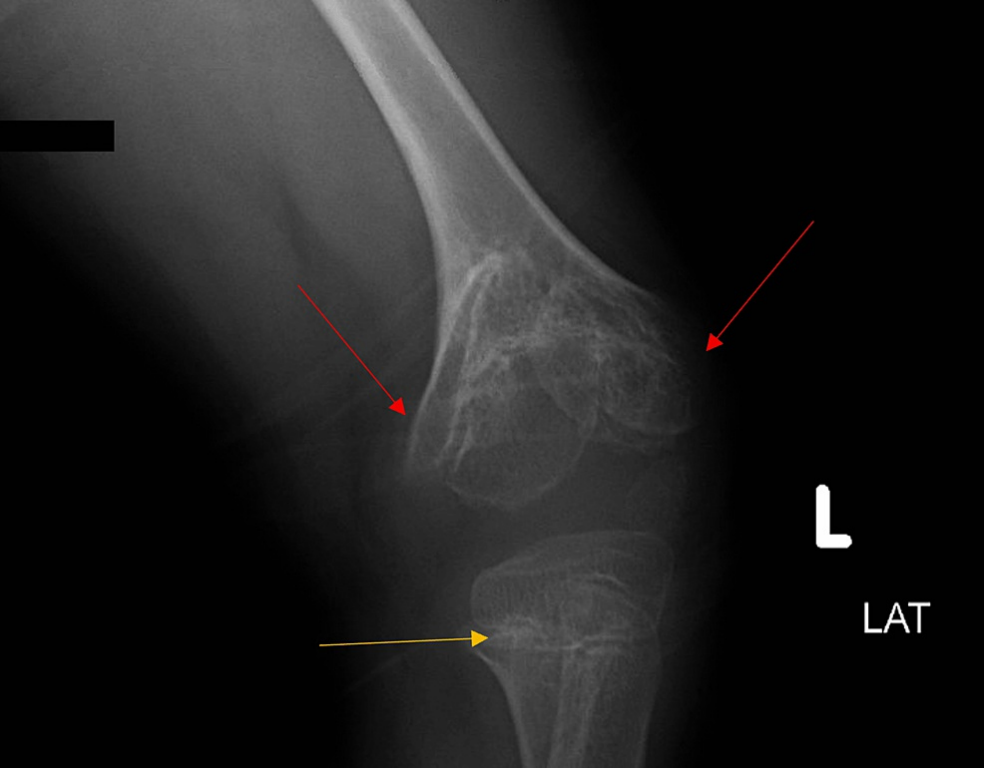
An X-ray of the left knee (lateral view) was performed in December 2021. In this figure, the colors illustrate the following: red: enlargement of the femoral condyles; yellow: sclerotic changes and central depression of the proximal tibial metaphysis.

In addition, hormonal and organ profiles for thyroid, parathyroid liver, and kidney functions were appropriate for her age. Genetic analysis including the chromosomal microarray analysis showed no positive results. For skeletal screening, a complete bone screening with an X-ray was performed, which showed an abnormality at only the knee sites. Other regions were biologically and anatomically accepted for her age. X-ray of the lower limbs (Figures 1, 2) illustrated bilateral knees with different views performed in December 2021. The medical report stated that there was length discrepancy (left > right) between both limbs with overall femur shortening, a cone-shaped deformity of distal femur metaphysis with a central fusion of the epiphysis, and enlargement of the femoral condyle, femoral neck widening, sclerotic metaphyseal change of the proximal tibia, and central depression in the proximal tibial metaphysis.

**Figure 3:**
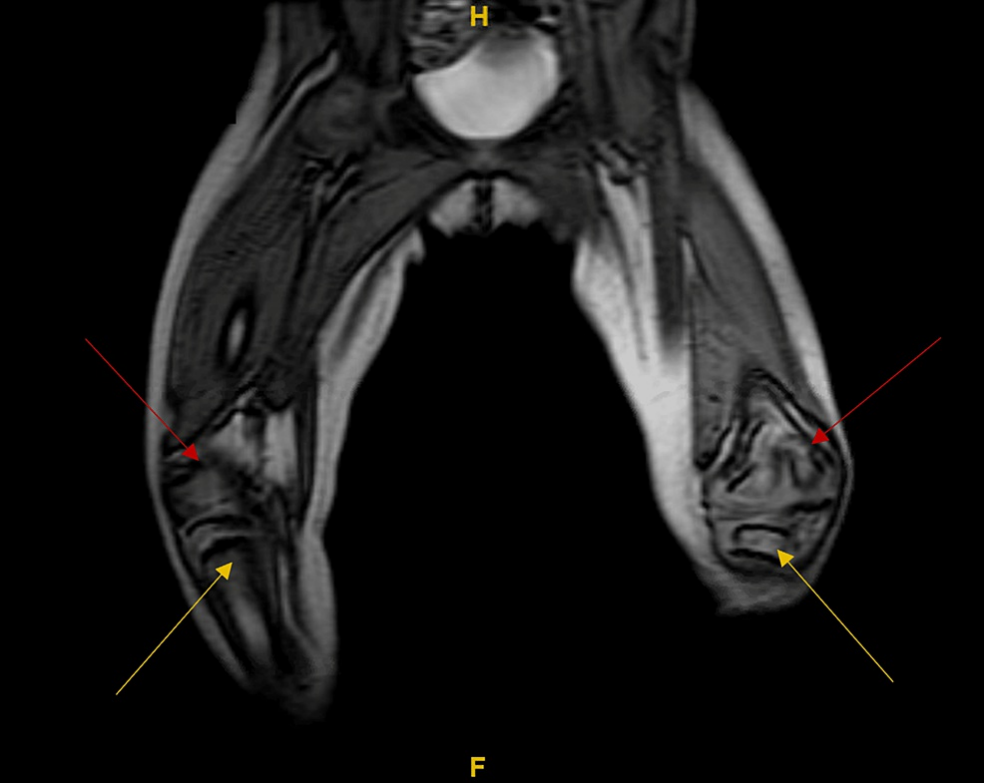
Magnetic resonance imaging of the bilateral lower limbs without contrast (coronal view) was performed in December 2021. In this figure, the colors illustrate the following: red: the distal metaphysis of both femurs was cone-shaped with corresponding deformed epiphysis and symmetrical narrowed growth plates; yellow: both proximal tibial metaphysis and epiphysis were minimally cone-shaped.

**Figure 4:**
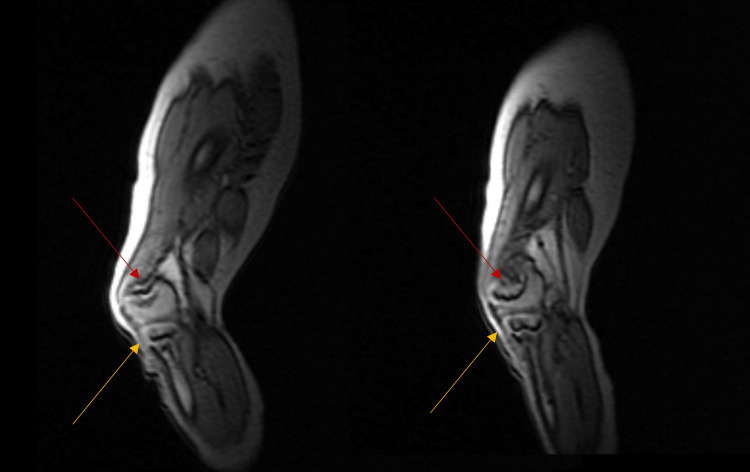
Magnetic resonance imaging of the left knee without contrast (sagittal view) was performed in December 2021. Red: the distal metaphysis of both femurs was cone-shaped with corresponding deformed epiphysis. In this figure, the colors illustrate the following: red: cone-shaped epiphysis and symmetrical narrow growth plates; yellow: both proximal tibial metaphysis and epiphysis were minimally cone-shaped.

In March 2022, the study was repeated and compared to the previous findings; however, no differences were found. Likewise, in December 2021, magnetic resonance imaging (MRI) of the lower limbs showed few abnormalities. First, the distal metaphysis of both femurs was cone-shaped with corresponding deformed epiphysis. Moreover, both growth plates were narrowed yet symmetric. Similarly, both proximal tibial metaphysis and epiphysis were minimally cone-shaped (Figures 3, 4).

The established diagnosis was bilateral congenital deformity (bilateral bifid distal femur) with abnormal gait. After multidisciplinary discussion with pediatrics, genetics, pediatric neurology, and other orthopedic pediatric consultants, a treatment plan was established, which included the application of a Taylor spatial frame in the femur to gradually correct the deformity one side at a time. First by operating on the right limb to lengthen the femur by 3 cm, followed by the left side at a later age. However, the parents preferred to seek another expert opinion in this regard. Despite that, the patient will benefit from gradual deformity correction using a Taylor spatial frame in the femur.

## Discussion

Femoral bifurcation was first described by Ehrlich in 1885 in a case associated with tibial hypoplasia [2]. It is a rare defect, described as a part of the Gollop-Wolfgang complex [2]. The Gollop-Wolfgang complex is a congenital orthopedic malformation seen in 1/106 live births, characterized by a distal femoral duplication and tibial agenesis with or without hand ectrodactyly [3]. In 1976, Ogden suggested that splitting of the cartilaginous femoral anlage early in development and subsequent partial failure of the proximodistal induction of the hindlimb explain the bifurcation and the association of tibial hemimelia, as cited in Ostrum et al. [1]. According to Lewin and Optiz in 1986, as cited in Cakir et al., the growth of the lower limb is controlled by two developmental fields, namely, the tibia and fibula. The tibial developmental field controls the development of the distal femur, tibia, and hallux. Thus, a defect in this field results in distal femur duplication, tibia agenesis, and pre-axial polydactyly or ectrodactyly [2]. However, isolated femur bifurcation is significantly rare and was first reported in 1987 by Ostrum et al. [1]. Following thorough investigations, this patient demonstrated bifurcation of the femoral head with no corresponding significant anomaly in the tibia or fibula, in contrast to the previously described cases of distal bifurcation of the femur associated with tibial hemimelia and fibular agenesis. As suggested earlier, femoral bifurcation and other congenital anomalies such as heart defects are associated with the proximal chromosome 8q, which may be involved in lower limb development [2]. The patient was a healthy girl with no mental, developmental, or other systematic abnormality except for the bifurcated short femur and length discrepancy between lower limbs resulting in abnormal gait. Likewise, the first case had an isolated bifurcated femur that presented with only cosmetic concerns [1]. Comparing their findings with this case corresponds with their reasoning of what was explained to be not a result of ectopic tibial anlage and disagrees with the Ogandas theory.

On reviewing the literature, there was no single best approach for the treatment of such cases. In 1987, a case of a boy who presented with knee contracture and distal femur bifurcation was reported, similar to this case. First, for the progressive flexion contracture, a posterior release of his right knee was performed at the age of nine months to improve the 60-degree flexion contracture. Then, a trial of distal femur osteotomy was attempted to fuse the condyles together, yet the femur regressed to its original state after three years [1]. In another case, a seven-year-old boy presented with bilateral tibial hemimelia and left femoral bifurcation. The management involved the resection of the anteromedial limb of the bifurcated femur and disarticulation of the knee at the end of the posterolateral limb [4]. In another case of a 12-year-old male with a bifurcated femur and complete ipsilateral complete hemimelia of the tibia, excision of the bifurcated femur, knee disarticulation, and prosthetic fitting gave a good early outcome [5]. Thus, a multidisciplinary team is needed to decide the approach to achieve the best outcomes for similar cases. In this presentation, a gradual deformity correction using a Taylor spatial frame in the femur was recommended. In addition, the other head of the femur can be used as a graft in this procedure. This choice of treatment was not available in our hospital; hence, the patient was directed to another facility and was offered to seek another opinion if desired.

## Conclusions

Our patient is a three-and-a-half-year-old girl who presented with abnormal gait and bilateral knee contractures for which further diagnostic studies have been performed. The X-ray scan showed femoral bifurcation and leg length discrepancy and MRI demonstrated similar findings and growth plate narrowing. After discussion with multidisciplinary teams of different specialties, gradual deformity correction using a Taylor spatial frame in the femur has been suggested; however, the patient did not complete her care with us. Outcomes in this rare condition can be hardly decided upon. As a result, reported cases with various presentations had different management plans. Further research and case reporting are essential to better define the pathogenesis, prognosis, and treatment of this condition to improve the morbidity of such cases.
